# Long-term follow-up of donor site morbidity after defect coverage with Integra following radial forearm flap elevation

**DOI:** 10.1007/s00238-013-0918-0

**Published:** 2014-01-10

**Authors:** Anna Wirthmann, Juliane C. Finke, Pietro Giovanoli, Nicole Lindenblatt

**Affiliations:** Division of Plastic and Reconstructive Surgery, Department of Surgery, University Hospital Zurich, Raemistrasse 100, 8091 Zurich, Switzerland

**Keywords:** Radial forearm flap, Integra, Reconstruction, Donor site morbidity, Tendon coverage

## Abstract

**Background:**

The radial forearm flap (RFF) is known for its excellent and reliable results regarding defect coverage though donor site morbidity represents a persisting problem. Integra is widely used in reconstructive surgery. This study aims to assess long-term outcomes regarding functionality and cosmesis after donor site coverage with respect to esthetic appearance, scar quality, and wrist function as well as ability to work after donor site coverage with Integra and split-thickness skin.

**Methods:**

The prospective follow-up exam, after a mean time of 23.8 months, enrolled 13 patients. Identification of patients’ comorbidities and evaluation of the subjective esthetic outcome, sensibility, cold intolerance, and scar instability was assessed by a questionnaire. In clinics, designed Pinch test assessed scar tissue mobility over the flexor tendons. The Vancouver Scar Scale (VSS) was calculated; wrist function and grip strength were determined.

**Results:**

The satisfaction score for the esthetic appearance of the donor site was in average 3.42 ± 0.44. Two patients experienced an unstable scar and cold intolerance. The VSS resulted in a value of 4.2 representing a good result. The pinch test revealed an average scar mobility of 8 mm without any tendon adhesions. Active range of motion of the wrist was equal to the non-operated site as was grip strength. The pinch test showed a significant negative correlation with the VSS making it a reliable tool to measure scar quality.

**Conclusion:**

Long-term results show an esthetic and functional successful defect coverage of the RFF donor site by the use of Integra and split-thickness skin.

Level of Evidence: Level IV, therapeutic study.

## Introduction

The radial forearm flap (RFF) is one of the most commonly applied flaps in plastic surgery. Since its introduction in 1981, it has evolved to be the standard free flap for defect coverage in head and neck tumor surgery [1, 2]. Next to this, it can be applied as a free or pedicled flap for several indications including reconstruction of upper extremity defects or phalloplasty and can be raised as a pure fasciocutaneous or a composite flap including bone, muscle, nerve, or tendon [3, 4]. The main features of the RFF are thin and often hairless skin, pliability as well as a large and reliable pedicle. However, despite the excellent results regarding defect coverage, donor site morbidity represents a persisting problem, which needs to be further addressed in order to improve overall outcome. Defect coverage of the donor site typically is achieved by application of a split-thickness skin graft (STSG). Often, these grafts do not heal properly resulting in exposed tendons, prolonged wound healing, and even reoperations [5]. Skin graft loss in major studies has been reported to be as high as 28 % [6]. Moreover, STSG use for defect coverage often may lead to tendon adhesion, reduced range of motion, and poor cosmesis [7]. Several efforts have been made to lower donor site morbidity including modification of dissection techniques, coverage with full-thickness skin grafts (FTSG), local flaps to achieve primary closure, or avoiding the flap in total and choosing free flaps with less donor site morbidity instead [8, 9].

Soft tissue coverage with Integra dermal regeneration template has been successfully used in reconstructive and burn surgery to increase the thickness of the resulting skin and to cover underlying structures like bone or tendon [10]. However, it requires a two-stage procedure before final defect coverage to allow for adequate vascularization of the template [11]. Integra provides an immediate donor site coverage independent of wound size [12]. As an additional benefit, it creates a gliding surface between the underlying structures, e.g., paratenon or nerves and the STSG. Despite these beneficial properties, it has been investigated in the past only in two short-term clinical studies to cover the RFF donor site with satisfactory immediate results [7, 13]. However, no long-term outcomes have been reported so far.

Therefore, the aim of this prospective follow-up study was to investigate the long-term outcome with respect to esthetic appearance, scar quality, and wrist function as well as ability to work after donor site coverage following RFF elevation with Integra and STSG.

## Material and methods

### Integra dermal regeneration template

Integra dermal regeneration template (Integra Life Sciences, Plainsboro, NJ, USA) is a template consisting of an outer silicone layer and an inner dermal replacement layer, consisting of cross-linked type-1 bovine tendon collagen coated with glycosamino-glykan. The chondroitin-6-sulfate serves as a three-dimensional pattern like normal dermal fibers. This inner layer acts as a tissue scaffold for dermal cellular ingrowth and remodeling without contraction and scarring. Meanwhile, the outer silicone layer imitates epidermal skin acting as a protective fluid barrier [7].

### RFF harvest and coverage of the donor site

**Fig. 1:**
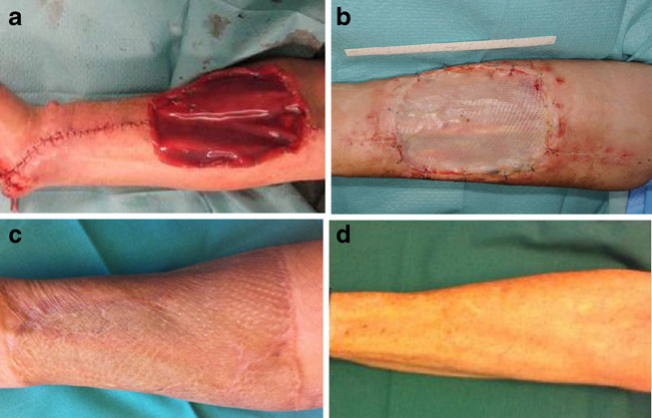
Defect coverage with Integra: **a** intraoperative view, **b** 3 weeks postoperative with applied STSG, **c** 24 months postoperative, **d** 51 months postoperative. Pictures were taken from different patients

Patients underwent RFF elevation in a subfascial plane preferably from the non-dominant arm. Briefly, the Allen test was performed preoperatively. The flap was outlined according to the size of the defect. Dissection then was started from the ulnar side, including the fascia and deepened toward the radial artery. Special attention was given to leave the paratenon of the flexor tendons intact. The donor site then was downsized partly by primary closure of the defect. On the residual defect site, an Integra sheet of respective size was attached with clips and a tie-over dressing was applied (Fig. 1a). After 3 weeks and achieved vascularization of the Integra, a STSG from the thigh was applied in a second intervention (Fig. 1b). Immobilization of the forearm and wrist in a palmar splint was performed for 3–4 weeks until complete healing had been achieved.

### Study design

The study was designed as a prospective follow-up study. In addition, data analysis was performed in a retrospective fashion from the patients’ data sheets and reports. Charts of adult patients of the Department of Plastic and Hand Surgery at the University Hospital of Zurich, Switzerland, who underwent RFF elevation between 2005 and 2011 were reviewed. Before 2008, donor site defect coverage was achieved by the application of STSG alone. In total, 40 patients received defect coverage with STSG alone or Integra and STSG. Patients who received defect coverage by STSG did not meet the inclusion criteria. Four patients were deceased by the time of follow-up; for three patients, no valid address was found in the data base and four other patients were in a state of poor health and therefore not able to participate.

Exclusion criteria were preoperative disabilities at the donor site extremity, minors, patients with clotting disorders, and non-German speakers. After receiving approval of the cantonal ethics committee, 23 patients who received coverage by Integra and STSG received an array by mail, plus a letter, outlining the purpose of the study and informing them that the participation was voluntary. A total of 13 patients enrolled to follow-up and provided verbal and written informed consent.

### Patient questionnaires

The donor site morbidity was estimated by a patient questionnaire concerning patients’ satisfaction with the esthetic outcome, sensibility, cold sensitivity, and instability of the scar. The esthetic result was judged by the patients at the time of the examination on a scale from one to five based on the following categories: 5, very satisfied; 4, somewhat satisfied; 3, neither satisfied nor dissatisfied; 2, somewhat dissatisfied; 1, very dissatisfied. Instability of the scar was defined by recurring ulceration after minimal trauma, e.g., wearing a sweater or a watch on the operated forearm. Additionally, patients were questioned concerning a distracting cold intolerance of the scar, problems to resume their original profession after the operation, and the payment of a disability pension.

A second questionnaire aimed at data acquisition about risk factors for poor wound healing (medication with ASS, steroids, immunosuppressants, nicotine abuse, and systemic disease) as well as obtaining information on risk factors for poor scarring, such as gender, race and age, performance, and duration of scar massage as well as application of silicone sheets. Previous operations on the respective arm and pre-existing limitations of function were recorded.

### Clinical examination

**Fig. 2:**
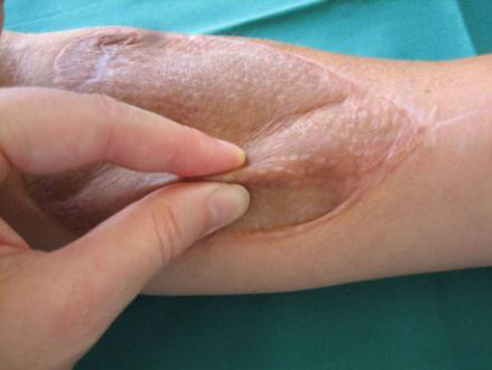
Pinch test: a skin fold is lifted 1 cm from the lateral and 1 cm from the medial border. Values are added and the sum is divided by two to yield the final score

A clinical examination between the operated and the non-operated arm was conducted during the follow-up examination. Scar quality was determined by the examiner based on the Vancouver Scar Scale (VSS) divided into the subgroups pigmentation, vascularity, pliability, height, pain, and itching [14]. All scars were photographically documented (Fig. 1c, d). Scar pliability was measured by the pinch test (Fig. 2). The pinch test was standardized by lifting a skin fold 1 cm from the lateral and 1 cm from the medial border and measuring its height. The sum was divided by two. A value of 5 mm or greater represented a good tissue laxity including the ability to separate the skin from underlying structures. Sensibility was determined by two-point discrimination (2 PD) at four different areas of the scar. A 2 PD of 30 mm or less was rated as positive and the sensibility determined to be intact. Sensibility was rated to be partially intact of the 2 PD was less than 30 mm in more than 50 % of the tested spots.

Active range of motion (aROM) of the wrist with respect to extension/flexion and pronation/supination was determined. The non-operated side was set as a control. Active range of motion was defined with a goniometer. If the operated side showed a difference of more than 10° compared to the non-operated side, this was classified as pathologic. Additionally, grip strength of the hand and the thumb pinch test were performed. Assessment was done by the patient squeezing the examiners hands while applying the maximal possible force. Pinch or grip strength of the healthy collateral site was defined as possible maximal power to be achieved. Results were measured by establishing a scale from 0 to 5 according to the Medical Research Council scale for muscle strength in which the patients’ effort is classified as follows: 5, normal muscle contraction against full resistance; 4, reduced muscle strength and muscle contraction against resistance; 3, further reduced muscle strength, joint can be moved only against gravity with the examiner’s resistance completely removed; 2, muscle move if the resistance of gravity is removed; 1, trace or flicker of movement or fasciculations observed in the muscle; 0, no movement observed. The presence of any contractures of fingers and wrist as well as the presence of contracting scar bands was evaluated.

### Patient characteristics

Patient characteristics are shown in Table 1. Mean follow-up time was of 23.8 months (range 6–51 months). Nine out of 13 patients successfully underwent defect coverage of the head and neck after tumor resection. Three patients had undergone the creation of a phalloplasty, and one patient presented with a lower extremity soft tissue defect. Scar massage was performed by seven out of 13 patients. The mean duration was 7.9 months (range 0–12 months). A silicone sheet was applied only by three patients in average for 6.7 months (range 1–15 months).

**Table 1:** Patient characteristics

Group	Patients
	*n* = 13
Age	52.4 ± 4.7 years
Gender	
Male	7
Female	6
Race	
North European	10
South European	2
Asian	1
Medication	
ASS	1
Steroids	1
Immunosuppressants	0
Systemic disease	
aHT	5
pAVK	0
DM	1
Nicotine abuse	5.5 ± 3.2 py

Values are gives as mean ± SEM

*ASS* acetylsalicylic acid, *aHT* arterial hypertension, *pAVK* peripheral arterial artery disease, *DM* diabetes mellitus, *py* pack years

### Statistical analyses

After proving the assumption of normality and equal variance across groups, differences between groups were assessed using one-way ANOVA followed by the appropriate post hoc comparison test. Mann–Whitney *U* tests were applied for nonparametric distributions. Overall statistical significance was set at *p* < 0.05. Values are given as mean and range or mean and SEM. Correlation analysis was performed using the Pearson test. Statistics and graphics were performed using the software packages SigmaStat and SigmaPlot (Jandel Corporation, San Rafael, CA, USA).

## Results

### Patient satisfaction with esthetic outcome

The mean satisfaction score for the esthetic appearance of the donor site at the time of follow-up in our patient collective was 3.42 ± 0.44. There was no correlation between the score and the time after the operation; thus, scar maturity had no influence on the subjective esthetic judgment of the patients. Both patients with unstable scars rated esthetic outcome as value 1. Transsexual patients after creation of a phalloplasty showed a tendency towards less satisfaction with the esthetic appearance (2.5 ± 0.9 phalloplasty vs. 3.7 ± 0.5 defect coverage; *p* = 0.2). Correlation analysis showed no significant relationship between the subjective esthetic estimations of the patients and the VSS established by the examiner. Also, there was no correlation with the presence of cold intolerance or the donor site with respect to dominant or non-dominant hand. Next to this, there was no difference with regard to medication, nicotine abuses, or the use of scar massage and silicon sheets.

### Scar quality

**Fig. 3:**
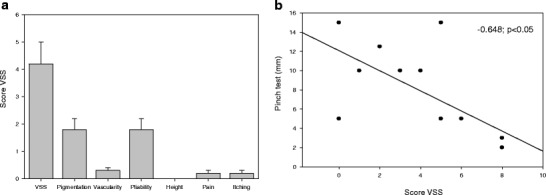
**a** VSS and subgroups, **b** regression analysis shows a significant negative correlation between VSS and pinch test score

After Integra and STSG application, donor sites in 12 out of 13 patients healed without problems over a time period of 4 weeks, and no exposure of tendons or necessary operative revisions of the donor site was reported. One patient reported an extended wound therapy over a time period of 9 weeks including regular presentations to the outpatient clinic. Defect closure could be finally achieved by conservative treatment. Scar quality assessed by the VSS is illustrated in Fig. 3a. The lowest possible score on this scale indicating the best scar quality, i.e., the quality of healthy skin is 0, the highest score is 22 [14]. In our patient, collective VSS averaged 4.2 (range 0–8) representing a good scar performance. No hypertrophic scars or keloids were observed. Three patients reported overall dissatisfaction with the scar (scores 1 and 2). Considering the single criteria, vascularity of the scar tissue showed the least favorable results (mean 1.8, range 0–3) representing a red to purple tissue color. Next to this, scar pliability showed an average number of 1.8 (range 0–4) representing a tissue quality between supple/flexible and yielding to pressure. All scars were flat going in line with the absence of any hypertrophic scarring and keloid formation, also not in the relatively young scars. Pain and itching at the scar site were of no major importance, and maximal values reached were occasional complaints in two patients per group.

The scar pinch test was in average 7.7 ± 1.3 mm with a range from 2 to 15 mm. This symbolizes a good movement of the tissue including the Integra neo-dermis over the underlying structures in particular tendons and nerves. This goes in line with the absence of major pain problems or movement restriction. The pliability of the grafted tissue corresponds with a low rate of scar contractures. A significant negative correlation between the pinch value and the VSS could be detected (*r* = −0.648; *p* < 0.05) (Fig. 3b). Therefore, the newly established method to estimate tissue mobility and pliability appears to be valid also to estimate the scar tissue in general.

No significant correlation between VSS and scar massage or the use of silicone sheets existed. However, patients with high VSS scores tended to have performed prolonged silicone sheet application, i.e., in two patients with a VSS of eight silicone sheets were utilized and scar massage was performed for 12 months. Interestingly, these two patients were the ones presenting with unstable scars at the follow-up.

**Fig. 4:**
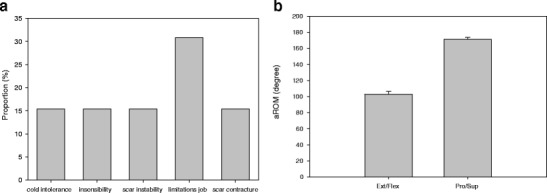
**a** Outcome after application of Integra and STSG, **b** aROM of the wrist

A feeling of cold intolerance at the operation site was reported by two patients (Fig. 4a). Two patients suffered from recurring ulceration at the donor site due to minimal trauma. Two out of 13 patients had a negative 2 PD test on the donor site of the operated forearm, 6 out of 13 a positive 2 PD, and five patients a partially intact 2 PD. Scar contractures consisting of single cords were seen in two patients with no functional limitations.

### Functional outcome

Mean aROM of the wrist was determined and showed no relevant impairment in all patients. aROM for extension/flexion of the wrist on the operated side was in average 103.8 ± 3.6° (range 75–115°) and showed a difference to the healthy side of more than 10° in four patients (Fig. 4b). In these four patients, aROM amounted to 92.5 ± 8.8° when compared with the other patients (108.9 ± 2.3°; *p* = not significant). Overall, aROM even in the least favorable cases was well above a functionally limitation extent, and patients did not complain about problems using the respective hand in daily activities.

aROM pronation/supination was 171.2 ± 2.5° in average (range 155–180°). Regarding pronation and supination, no patient showed any limitation of more than 10° compared to the healthy control side. Grip strength on the operated reached the maximum value of 5 in all patients and showed no limitations compared to the non-operated site. Thumb pinch strength was 4.9 ± 0.1 in average and found to be slightly decreased in only one patient. It should be mentioned that decrease in pinch strength was accompanied with a decrease in extension in this case. Two patients who had previous surgery on the forearm, namely osteosynthesis after radial fracture and a carpal tunnel release did not experience any functional limitations. No relationship between wrist function and esthetic outcome, VAS, or pinch test was detected.

### Ability to work

Collected data revealed that 11 of our collective of 13 were employed at the time of surgery. Four out of 11 patients reported a delayed re-entry into employment. Two of those patients worked as car chauffeurs, one as a social worker and one as a referee. One patient working as a farmer had to undergo a vocational retraining. One patient was not able to work anymore at all and received a pension. Especially in those cases where the RFF has been harvested for the creation of a phalloplasty, patients undertook strong efforts to hide the scar on the forearm.

## Discussion

In the past 20 years, the RFF has become a standard free flap in microsurgery with numerous indications and excellent results with respect to closure of the primary defect [5]. However, the price of a considerable functional and esthetic morbidity of the weakened donor side often has to be paid [9]. Skin graft loss in major operative series has been as high as 16 to 28 % and implies exposed flexor tendons, prolonged healing times, or necessary reoperations to close the donor site defect [6, 15]. This is particularly of interest since the patients’ health conditions receiving defect coverage by RFF usually is quite poor. Plastic surgeons may face an immunosuppressed patient, undergoing radiation, and/or chemotherapy. Therefore, there is a need for a safe and reliable method of donor site defect coverage.

Numerous attempts to reduce donor site morbidity have been made in the past decades. An ulnar artery perforator flap reliably covered defects up to 12 × 6 cm [16]. Karimi et al. recommended a closing technique with a purse string suture and covering the residual defect with a STSG [3]. When defect coverage is performed with STSG, the chance of providing a gliding surface for a functional tendon without adhesions is low. In the presented study, 12 out of 13 donor sites healed within the usual time frame of 4 weeks, and no tendons or other structures were exposed within the wound. However, this result also may be achieved with a meticulous dissection technique leaving the paratenon intact and thus providing a favorable wound bed even for a STSG alone. To avoid the donor site morbidity after skin harvest, STSG autologous skin equivalents can be used on top of Integra with good functional and esthetic results even though skin color is not matching [17]. As an alternative, the STSG can be harvested from the primary flap [18].

Next to this, no patient of the presented study experience tendon adhesions, nerve irritations, pain, or presented with a significant loss in aROM of the wrist and hand. In line with Shores et al. [12], we did not witness any case of acute complications, i.e., infection of the Integra template or significant skin graft loss during incorporation. Only one patient experienced initial partial take of the STSG over the flexor tendon requiring extended observation and wound therapy for 9 weeks.

Davis et al. compared STSG and FTSG for donor site coverage [19]. In this study, donor site healing times were proven to be similar for STSG and FTSG under standardized conditions including a general satisfaction with donor site scars in both groups. Even though a FTSG is a reliable option for defect coverage, defect size is a major limitation. Additionally, the rate of FTSG take reported in literature between 84 and 88 % is typically lower than published rates of 84–98 % for STSG [7]. Poor graft taking of FTSG often results in hypertrophic scars, which is a common esthetic concern in patients. Also, FTSG donor site morbidity cannot be neglected [7]. Integra can be easily outlined at the size of the defect and therefore represents a safe and reliable way of defect coverage with reproducible results. Thus, by combination of Integra and a STSG, a full-thickness skin can be re-created. This goes in line with the results of our study. The VSS yielded a favorable average value of 4.2 taking into account that also patients who were operated only 6 months ago were included into the study. The pinch test showed a satisfactory movement of the scar over the tendons of 8 mm on both sides of the scar in average and no adhesions to the underlying tissue were noted. According to the study of Ito et al. who analyzed “what the patient really bothers” the skin depression by using FTSG was the most concerning (30 %) to patients [8]. STSG are even thinner than FTSG, so it can be assumed that the overall esthetic acceptance is even lower.

Defect coverage by Integra and STSG requires a two-stage procedure, which can be regarded as a disadvantage by the patient. Jacob et al. compared one stage donor site defect coverage by Alloderm and STSG vs. STSG alone. Their results showed superior cosmetic results in the Alloderm and STSG group than in the control group, as well as in terms of tendon exposure, functional impairment, and paresthesia without a significant difference [20]. The procedure can be regarded as an alternative to defect coverage by Integra as a two-stage procedure. A two-stage procedure requires a prolonged immobilization. As an alternative single layer, Integra can be used in a single stage procedure as done by Demiri et al. with satisfactory functional results, overall take rate (95–98 %), and pliability as well as overall appearance of the reconstructed areas (mean Vancouver Scar Scale Score 1.875) [21].

To minimize donor site morbidity further, we have adopted a careful dissection technique. The remaining paratenon provides a well-vascularized recipient bed for the Integra template. Another possibility to even reduce tendon exposure may be the preservation of the deep forearm fascia [22]. Results of previous research are ambiguous: a significant limitation in range of motion at the harvesting site [23] is shown in some and disproven in other studies [12]. Kamal et al. showed equal or better clinical outcomes by adopting the suprafascial dissection technique compared to the infrafascial with regard to tendon adhesion (0 vs. 0) and sensory nerve damage at donor site (0 vs. 2), with an equal number of flap failure (1/1) [24]. When comparing defect coverage of a suprafascial dissection technique by FTSG to STSG plus a negative wound pressure, initial graft taking is as high as 96 % in 5 days and 100 % in a month [25]. However, Lutz et al. showed five cases out of 95 with partial skin graft failure when coverage of the suprafascial defect is performed by FTSG or STSG alone. In those cases, grip power and pulp-to-pulp pinch power is significantly decreased [26].

With respect to functionality, our results demonstrate favorable functional outcomes. We did not observe any limitations in grip strength or wrist movement at the donor site. Four patients showed a not significantly decreased aROM of more than 10° difference when compared to the healthy side. In literature review, similar results are found when using FTSG on the reconstruction site [7, 8]. If defect coverage was performed with STSG, cases of tendon adhesion are reported as high as 19–33 %.

While initially the patients’ main postoperative concern is the reconstruction site, the donor site becomes more important to the patients after approximately 1 year [8]. This may explain why only seven of the 13 patients performed scar massages and only three out of 13 used a silicone sheets to improve scar quality.

It is to be noticed that the mean satisfaction score after a mean postoperative time to follow-up of 23.77 months is as high as 3.42 out of a maximum of 5. However, still three patients were dissatisfied with their scar and reported a score of 1 or 2. Studies comparing STSG to other options of defect coverage report high dissatisfaction score in up to 39 % of patients [7]. Previous research also shows a higher dissatisfaction score in female patients [23]. In comparison to these data, patients treated with Integra and STSG in our study appear to have a higher satisfaction with the esthetic results overall.

Various publications show that defect coverage by STSG alone often results in poor wound healing, with a persisting instability of the scar. Graft failure and tendon exposure rates are reported as high as 30 to 50 % in several series [27]. According to our literature research, there has been no long-term follow-up after RFF elevation with regard to instability of the scar reported. However, our study shows scar instability in only two patients when defect coverage is performed with Integra and STSG. This may at least in part be due to the high proportion of sensible scars in our patient collective. Next to this, the thicker resulting neo-dermis most likely leads to a more stable and less vulnerable scar. Even though the benefits of the Integra scaffold are obvious, the additional costs of approximately EUR 1,900 per piece of 10 × 12.5 cm have to be taken into account. Even though this adds to the total cost of the operation, a better long-term outcome and a quicker recovery will reduce costs in long-term.

In four patients of our study group, the RFF served either for the construction of a phallus in the process of sex change or reconstruction after a traumatic soft tissue defect. They presented to surgery as healthy individuals without any mentionable comorbidities, with an average age of 37 years. Those patients had a higher resentment in general about the anesthetic appearance on the forearm than tumor patients.

In conclusion, our data show that defect coverage of the donor site after RFF elevation can be successfully achieved by Integra and STSG with stable and satisfactory long-term esthetic and functional results. Even after suprafascial dissection of the radial flap, it is advisable to perform donor site defect coverage with a skin equivalent to avoid depression of the defect site, especially for patients with high expectations regarding the esthetic outcome.
